# Accelerated motion corrected three‐dimensional abdominal MRI using total variation regularized SENSE reconstruction

**DOI:** 10.1002/mrm.25708

**Published:** 2015-05-21

**Authors:** Gastao Cruz, David Atkinson, Christian Buerger, Tobias Schaeffter, Claudia Prieto

**Affiliations:** ^1^ King's College LondonDivision of Imaging Sciences and Biomedical EngineeringLondonUnited Kingdom; ^2^ Centre for Medical ImagingUniversity College LondonLondonUnited Kingdom; ^3^ Philips Research HamburgHamburgGermany; ^4^ Pontificia Universidad Católica de Chile, Escuela de IngenieríaSantiagoChile

**Keywords:** motion correction, nonrigid motion, abdominal MRI, undersampling, total variation

## Abstract

**Purpose:**

Develop a nonrigid motion corrected reconstruction for highly accelerated free‐breathing three‐dimensional (3D) abdominal images without external sensors or additional scans.

**Methods:**

The proposed method accelerates the acquisition by undersampling and performs motion correction directly in the reconstruction using a general matrix description of the acquisition. Data are acquired using a self‐gated 3D golden radial phase encoding trajectory, enabling a two stage reconstruction to estimate and then correct motion of the same data. In the first stage total variation regularized iterative SENSE is used to reconstruct highly undersampled respiratory resolved images. A nonrigid registration of these images is performed to estimate the complex motion in the abdomen. In the second stage, the estimated motion fields are incorporated in a general matrix reconstruction, which uses total variation regularization and incorporates k‐space data from multiple respiratory positions. The proposed approach was tested on nine healthy volunteers and compared against a standard gated reconstruction using measures of liver sharpness, gradient entropy, visual assessment of image sharpness and overall image quality by two experts.

**Results:**

The proposed method achieves similar quality to the gated reconstruction with nonsignificant differences for liver sharpness (1.18 and 1.00, respectively), gradient entropy (1.00 and 1.00), visual score of image sharpness (2.22 and 2.44), and visual rank of image quality (3.33 and 3.39). An average reduction of the acquisition time from 102 s to 39 s could be achieved with the proposed method.

**Conclusion:**

In vivo results demonstrate the feasibility of the proposed method showing similar image quality to the standard gated reconstruction while using data corresponding to a significantly reduced acquisition time. Magn Reson Med, 2015. © 2015 The Authors. Magnetic Resonance in Medicine published by Wiley Periodicals, Inc. on behalf of International Society for Magnetic Resonance in Medicine. This is an open access article under the terms of the Creative Commons Attribution License, which permits use, distribution and reproduction in any medium, provided the original work is properly cited. **Magn Reson Med 75:1484–1498, 2016. © 2015 The Authors. Magnetic Resonance in Medicine published by Wiley Periodicals, Inc. on behalf of International Society for Magnetic Resonance.**

## INTRODUCTION

Respiratory motion is a major source of artifacts in abdominal imaging, causing ghosting and blurring in the reconstructed image 1. In two dimensions, a simple approach to reduce these artifacts is to acquire the image during breathhold(s). Unfortunately, breathholding is difficult in three dimensions without compromising resolution/scan time; therefore, a three‐dimensional (3D) free‐breathing acquisition is desired. Free‐breathing acquisition is usually performed using respiratory gating techniques 2. Respiratory gating monitors the position of the diaphragm in the superior–inferior (SI) direction using external sensors, navigator echoes or self‐gating. External sensors require additional preparation and provide only a relative measurement of diaphragm displacement 3. Navigator echoes do not have these limitations, but can interfere with image acquisition (e.g., disrupting the balanced steady state free precession acquisition) 4. Self‐gating techniques 5 extract motion information from the acquired data itself, in general using the central k‐space profile. Respiratory gating only accepts data within a small gating window, minimizing motion artifacts at the expense of additional scan time. Moreover, in subjects with highly irregular breathing patterns, drift in respiratory motion can lead to scan termination due to low scan efficiency. Respiratory gating monitors SI translational motion, although it is known that abdominal acquisitions are corrupted by large nonrigid components of motion 6, 7, 8.

In cardiac imaging, several approaches have been proposed for motion compensation. In 9, SI motion is estimated by means of self‐navigation, enabling the estimation of data weighting factors from the distance to a reference respiratory phase. This information is then used in a weighted iterative reconstruction with total variation regularization to reduce respiratory artifacts. More complex 3D affine motion has been estimated from 3D low resolution image navigators and used to correct the actual acquisition in image space 10. An alternative approach 11 estimates 3D affine motion from respiratory resolved images and uses it to correct the acquired k‐space before reconstructing the image. In Prieto et al 12, motion is estimated similarly, but is then corrected directly in the Cartesian reconstruction using the General Matrix Description (GMD) 13. Additional solutions have been proposed to correct the more complex nonrigid abdominal motion. A pixel‐by‐pixel translation correction has been used to correct nonrigid motion 14 using local autofocus 15. This technique requires the acquisition of additional navigator echoes as well as triggering and gating of a portion of the acquired data. A self‐gated motion corrected approach is proposed in Buerger et al 16, where nonrigid motion is estimated from undersampled respiratory resolved images. A motion compensated image is then obtained by warping all undersampled images to a common respiratory position. However, this image‐based approach has two major limitations: (i) aliasing artifacts of each undersampled motion state are warped to the common respiratory position and may persist in the final image, and (ii) the averaging of multiple motion states may lead to blurring in the final image. The latter effect has been shown for abdominal and cardiac images in 17, 18 when compared with the GMD approach. GMD corrects motion directly during the reconstruction process; although a previous estimation of the motion is required. This motion has been estimated from low‐resolution training acquisitions 19 or from a computationally expensive coupled motion reconstruction and motion estimation problem based on external sensors 20. These approaches have been demonstrated for fully and over‐sampled acquisitions only. Here, we propose a highly accelerated GMD‐based method to correct nonrigid motion in undersampled 3D isotropic abdominal images.

The motion estimation step of the proposed approach is an extension of the one used in 16 to account for undersampled acquisitions. Data are acquired under free breathing with a self‐gated golden‐radial phase encoding (G‐RPE) trajectory 21, 22, enabling the reconstruction of highly undersampled images at various motion states (respiratory bins). Unlike Buerger et al 16, where iterative SENSE reconstruction 23 was sufficient to independently reconstruct the bins, here we propose to reconstruct all motion states simultaneously using a spatial and temporal total variation regularized iterative SENSE (TV‐SENSE) approach, allowing reliable motion estimation from bins with higher undersampling factors than those reported in 16. Additionally, in the proposed method motion compensation is performed directly in the reconstruction, incorporating parallel imaging information and spatial total variation regularization (TV‐GMD). The proposed approach was tested on nine healthy volunteers and compared against a standard gated reconstruction.

## METHODS

**Figure 1:**
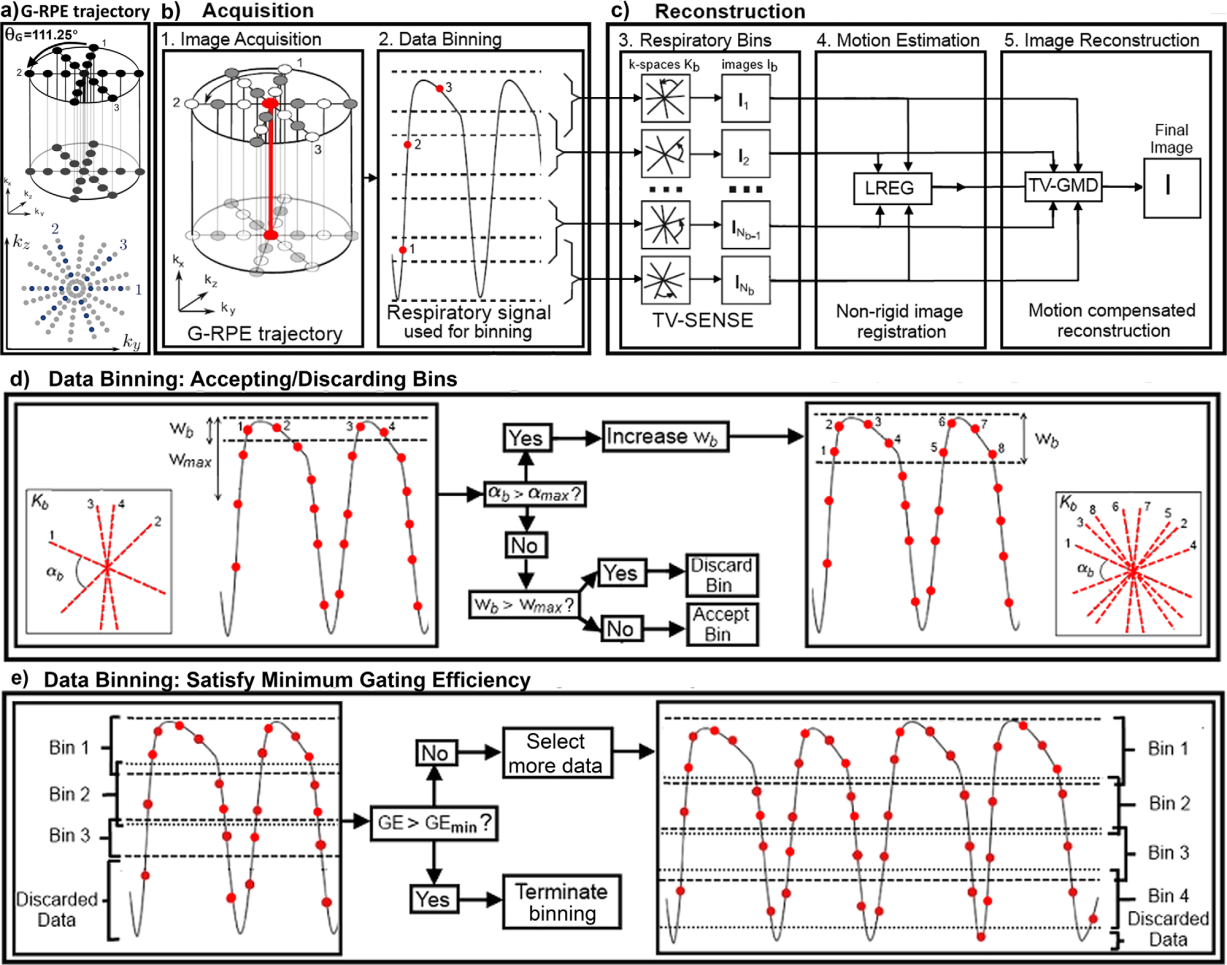
**a**: Top: Diagram of the golden radial phase encoding (GRPE) trajectory. Cartesian readouts (k_x_ direction) are acquired with a radial order in the phase encoding plane (k_y_ – k_z_). Consecutive radial profiles (numbered 1, 2, and 3) are separated by the golden angle *θ_G_* = 111.25°. a: Bottom: Diagram of GRPE undersampling in the phase encoding plane. Angular undersampling is achieved by skipping complete radial profiles (full gray radial profiles), while radial undersampling is performed along each radial profile by uniformly skipping readouts (gray readouts along radial profiles 1, 2, and 3). **b,c**: Diagram of the proposed approach in five steps. The acquisition is comprised of two parts: step 1. Image is acquired with 3D G‐RPE; step 2. The central k‐space line yields a respiratory signal which is used to bin data. The reconstruction is divided into 3 parts: step 3. Binned datasets are simultaneously reconstructed with TV‐SENSE; step 4. Motion is estimated by image registration (LREG) of the binned reconstructions; step 5. The estimated motion is used in the motion compensated reconstruction. Only the acquired data are needed to produce a motion compensated image. Adaptive data binning is performed in two steps: **d**: Each bin is initialized with a window size *w_b_*. If α_b_ is larger than α_max_, *w_b_* is increased, providing additional data for this bin. This process is repeated until α_max_ is respected. If the final *w_b_* is larger than *w_max_*, the bin is discarded; otherwise it is accepted. **e**: Insufficient data cause a significant fraction of bins to be discarded, leading to a poor gating efficiency (*GE*). If *GE* is smaller than *GE*_min_, an additional radial profile is considered and the full dataset (including the newly considered radial profile) is re‐binned. This process terminates when the *GE*_min_, α_max_ and *w_max_* constraints are simultaneously respected.

The proposed framework can be divided into five steps (Figs. 1b,c). In the first step, data are acquired with a self‐gated 3D golden‐radial phase encoding (G‐RPE) trajectory during free breathing. These data are sequentially assigned into different motion states, yielding a set of highly undersampled k‐spaces *K_b_* at the completion of the acquisition (step 2). Each bin is reconstructed with TV‐SENSE (step 3), producing undersampled respiratory resolved images *I_b_*. In the next step, nonrigid motion is estimated using image registration of *I_b_*. Finally, the estimated motion is used to reconstruct a motion corrected image using TV‐GMD.

### Image Acquisition

G‐RPE 21, 22 combines a regularly sampled Cartesian readout (*k_x_*) in SI direction with a radial phase encoding order in the *k_y_‐k_z_* plane, where radial profiles are separated by the golden angle *θ_G_* = 111.25° (Figure 1a, top). A G‐RPE acquisition can be undersampled radially by skipping readouts within each radial profile and angularly by acquiring fewer radial profiles (Figure 1a, bottom). This trajectory provides a quasi‐uniform k‐space distribution regardless of the number of profiles used 24, ensuring an optimal distribution of profiles in k‐space for different length of data acquisition. Additionally, the central k‐space readout (red line in Figure 1b) is acquired every N × TR seconds where is N the number of readouts within a radial profile and TR is the repetition time, allowing for respiratory self‐navigation. If the acquisition time for the radial profile (N × TR) is fast enough, respiratory motion within the radial profile can be considered negligible and the central k‐space readout can be used to navigate the entire radial profile.

### Data Binning

We identify the respiratory position of each radial profile by inferring the diaphragm displacement in the (SI) readout direction as described in Buerger et al 25. This self‐navigated respiratory signal is used to group the acquired data into different respiratory positions. During this process, referred as data binning, the radial profiles in each bin will not be equally spaced by the golden angle as they depend on the breathing cycle 16. Therefore a quasi‐uniform k‐space distribution is not guaranteed for any bin. k‐Space gaps may generate artifacts in *I_b_* and affect subsequent motion estimation. Thus, it is important to ensure that each bin has adequate coverage of k‐space for reliable motion estimation. The artifact level in *I_b_* is predicted using the maximum angle between two radial profiles (α), similar to 25. To guarantee that the image quality of *I_b_* is sufficient for reliable motion estimation we perform an adaptive binning that forces each bin to have α_b_ (maximum angle between two radial profiles in bin *b*) smaller than a predetermined α_max_ (determined empirically). To minimize intra‐bin motion, only bins with a bin window (*w_b_*) smaller than a maximum bin window (*w_max_*) are accepted.

Here, we perform an adaptive binning (Figs. 1d,e) with constraints to ensure reliable motion estimation. This process is controlled by 4 parameters: maximum overall undersampling factor (*R_max_)*, the maximum angle (α_max_), the maximum bin window size (*w_max_*) and the minimum gating efficiency (*GE*_min_). Gating efficiency (*GE*) is given by:

(1) $$GE=\frac{Accepted\;Profiles}{Total\;Profiles\;Acquired}$$

Respiratory bins are chosen such that within each bin, α_b_ < α_max_ (to limit reconstruction artifacts), and *w_b_* < *w_max_* (to limit intra‐bin motion). To limit undersampling artifacts in the final reconstruction of the complete dataset, a minimum amount of data to respect *R_max_* is enforced. A potential bin is first generated at the end‐exhale position with a bin window size equal to the image resolution. The bin window is increased until it has enough data to comply with the α_max_ constraint (Fig. 1d). If the bin window size exceeds *w_max_* the bin is discarded to minimize intra bin motion, otherwise it is accepted. A sliding window is used to initialize a potential bin in the next respiratory position and the process in Figure 1d is repeated.

Once the considered data thus far have been binned, the algorithm tests if the selected dataset satisfies the minimum gating efficiency. If the current gating efficiency is less than *GE*_min_ (Fig. 1e), then an additional radial profile is considered and the binning process is restarted (back to Figure 1d). Data binning terminates once enough motion states are adequately populated (α_b_ < α_max_, *w_b_* < *w_max_*) and the complete dataset satisfies the condition *GE* > *GE*_min_.

### Reconstruction of Respiratory Bins

The undersampled bins are simultaneously reconstructed with total variation regularized iterative SENSE (TV‐SENSE) 26, 27 given by:

(2) $${\hat{I}}_{b}=\;{arg\;min}_{I_{b}}{\left\{\|EI_{b}-K_{b}\right\|}_{2}^{2}+\lambda_{s}{TV}_{s}+\lambda_{t}{TV}_{t}\}$$

where ***K*** *_b_* is the acquired data at each bin *b*, ***E*** is the encoding matrix including coil sensitivities, gridding and Fourier transformation, ***I*** *_b_* the binned images to be reconstructed, ***TV_s_*** represents the 3D spatial total variation (intra‐bin) and ***TV_t_*** the temporal total variation in the respiratory direction (inter‐bin). The *l*_2_‐norm is the data consistency term. ***TV_s_*** is the sum of absolute differences in each bin; ***TV_t_*** is the sum of absolute differences between adjacent bins. Both of these transforms are *l*_1_‐norms operating in image space, defined as:

(3) $${TV}_{s}={\left\|\left\|\nabla_{s}I_{b}\right\|\right\|}_{1}$$

(4) $${TV}_{t}={\left\|\left\|\nabla_{t}I_{b}\right\|\right\|}_{1}$$

where ∇***_s_*** and ∇***_t_*** represent the 3D spatial gradient and the 1D temporal gradient, respectively. The parameters **λ_s_** and **λ_t_** define the balance between total variation regularization and data consistency. The ***TV_s_*** term removes noise‐like aliasing in the image and sharpens the main edges in the image, e.g., the diaphragm–lung boundary, allowing better image registration. Large **λ_s_** values enhance these effects at the expense of additional blurring (due to over‐smoothing). While blurring limits the accuracy of subsequent image registration, aliasing introduces errors in ***I*** *_b_* that can make the registration fail altogether. Hence, we use a large value for **λ_s_** to reconstruct artifact‐free respiratory bins and allow reliable motion estimation. The ***TV_t_*** removes additional aliasing in areas where there is little motion, like the central part of the liver. Large **λ_t_** values will introduce blurring in regions with large motion, e.g., diaphragm–lung boundary. Thus, we use small **λ_t_** values to distinguish motion states. Here, the ***TV_t_*** term amounts to a small regularization compared with ***TV_s_***, yielding minor improvements in the reconstructed image. The values for **λ_s_** and **λ_t_** were determined empirically by inspecting the output reconstructions of four training datasets covering the expected range of undersampling factors and respiratory amplitudes and choosing values that removed the most background aliasing without significantly blurring the image.

To accelerate bin reconstruction, a data consistency reconstruction (i.e. **λ_s_** and **λ_t_** set to zero) is used as the starting estimate for TV‐SENSE. This approach accelerates convergence and delivers a good approximation of the reconstructed image. These reconstructions are preconditioned 23 by intensity correction (from coil sensitivities) and a Voronoi based density compensation function 28, as our data acquisition (G‐RPE) is non‐Cartesian.

### Motion Estimation

A 3D nonrigid respiratory motion model is obtained from image registration of reconstructed bin images to a common respiratory position. This is done using the LREG tool 29, which performs an intensity based registration through a succession of local affine registrations, starting from a global affine down to small image blocks. The resulting deformation field is thus nonrigid.

### Image Reconstruction

The final undersampled image is reconstructed using a spatial total variation regularized GMD approach, based on the formalism introduced in Batchelor et al 13. The GMD approach is described by:

(5) $$K=\sum_{b}A_{b}FS_{c}U_{b}I=EI$$

where ***I*** is the ideal (motionless) image, ***U_b_*** are the spatial transformations for bin *b* obtained with LREG, ***S_c_*** are the coil sensitivities for coil *c*, ***F*** is the forward Fourier transform, ***A_b_*** is a logical matrix that selects the k‐space lines acquired for bin *b*, and ***K*** is the acquired multi‐channel k‐space data. Equation (5) is in the reference frame of the coils, where they are assumed to remain static. ***I*** may be obtained by inverting the encoding operator ***E***. Practical inversion of ***E*** is obtained with iterative methods such as the conjugate gradient (CG). The CG requires a symmetric positive‐definite matrix, thus we apply the method to the equivalent normal equation:

(6) $$E^{H}K=\left(E^{H}E\right)I$$

where ***E^H^*** is the Hermitian transpose of ***E***.

The proposed approach uses undersampled data, which results in noise amplification in the reconstructed motion corrected images due to poor conditioning of the inversion. This happens for two reasons: first, if the object moves outside the field‐of‐view (FOV) then information is permanently lost; second, correcting for motion within the FOV may cause k‐space inconsistencies, causing some samples to overlap and creating k‐space gaps. This effectively increases the undersampling factor (R), which contributes to making Eq. [6] undetermined 30. Furthermore, in practice the motion is not known exactly and errors in motion estimation can further contribute to the poor conditioning of this problem. It has been shown how multiple channels improve the condition of the motion reconstruction problem for fully sampled images 30. To reduce the effect of poor conditioning in undersampled datasets we include spatial total variation regularization ***TV_s_*** in the GMD reconstruction (TV‐GMD), given by:

(7) $$\hat{I}={arg\;min}_{I}\{{\left\|\left\|EI-K\right\|\right\|}_{2}^{2}+\lambda_{s}{TV}_{s}\}$$

This is solved with a nonlinear conjugate gradient reconstruction using the solution to the nonregularized approach as a starting estimate, similar to the previous description of the bin reconstruction. In the TV‐GMD reconstruction, the ***TV_s_*** term is used to remove noise‐like aliasing with small **λ_s_** values. The **λ_s_** for the TV‐GMD was determined empirically by choosing a value that reduces the remaining artifacts without significantly affecting image sharpness.

### Data Acquisition

Nine healthy volunteers were scanned under free‐breathing on a 1.5 Tesla (T) clinical scanner (Philips Achieva, Philips Healthcare, Best, The Netherlands) using a 32‐channel cardiac coil. Written informed consent was obtained according to institutional guidelines. Data were acquired with a balanced steady state free precession sequence with the following parameters: 287 × 287 × 287 mm isotropic FOV, 1.75 × 1.75 × 1.75 mm isotropic resolution, (TR/TE) = 3/1.43 ms, flip angle 30°. During acquisition, each radial profile was uniformly undersampled in the radial direction by a factor of 2. For the resulting reconstructed matrix size of 164 × 164 × 164, we need 256 radial profiles to approximately fulfil the Nyquist criterion in the angular direction, assuming a quasi‐uniform profile distribution. During the scan, 820 radial profiles were acquired to guarantee all volunteers provided enough data for different reconstruction approaches, including the self‐gated reconstruction which has a low scan efficiency.

### Simulations

The proposed approach estimates motion from highly undersampled respiratory resolved bin reconstructions (*I_b_*). A pseudo‐random distribution of radial profiles is sampled at each respiratory bin, depending on the breathing pattern. This may result in large k‐space gaps that can introduce artifacts in the reconstructed images. Specifically, k‐space gaps affect the image point spread function by broadening the main lobe/increasing side lobe amplitude, which results in blurring/aliasing artifacts 24. Blurring and residual artifacts may hinder subsequent image registration, producing inaccurate motion fields. A simulation was conducted in one representative volunteer to study the relationship between the maximum angle between two radial profiles (α_max_) and the accuracy of motion estimation. An end‐inhale image (*F*) was reconstructed with 25 different numbers of radial profiles (*p*), from fully sampled (256 profiles) to highly undersampled (26 profiles). The obtained undersampled images (*F_p_*) were registered to a reference fully sampled end‐exhale image (*F_r_*). The obtained motion fields were then compared with the ground‐truth: the registration between *F_r_* and *F*. We measure the average displacement error in the motion field ε(α) similar to 25:

(8) $$\varepsilon \left(\alpha \right)=\frac{\sum_{1}^{N}\sqrt{{\left({\vec{U}}_{\alpha_{\left(Kp\right)}}\left(n\right)-{\vec{U}}_{Fr}\left(n\right)\right)}^{2}}}{N}$$

where *U*_α(Kp)_ is the motion field between *F_p_* and *F_r_*, *U_Fr_* is the motion field between *F* and *F_r_*, and the sum is taken over all the voxels (*N* being the total number of voxels in the image).

### Comparison with Image Based Motion Compensation

A preliminary study was performed to compare the approach described in Buerger et al 16, hereby referred as image‐based motion compensation (IMC) with the proposed TV‐GMD. To evaluate the effects of blurring and residual artifacts in motion estimation, bin reconstructions using the α_max_ value, obtained by means of simulations, were performed using iterative SENSE (as in IMC) and with the proposed TV‐SENSE. Motion fields between neighboring bins of representative volunteers were obtained by means of LREG. A comparison of motion estimation obtained from undersampled SENSE and TV‐SENSE bin reconstructions was made by computing their displacement errors to motion fields obtained from a dataset 3× oversampled in the angular direction (considered as ground truth). Additionally, all nine datasets were reconstructed with IMC, using the same amount of data as the TV‐GMD reconstruction. This resulted in a total of 160 ± 37 acquired profiles for both TV‐GMD and IMC reconstructions. IMC reconstruction was performed with iterative SENSE 23, taking approximately 1 h. IMC and TV‐GMD reconstructions were compared by means of measures of liver sharpness and gradient entropy. TV‐GMD reconstruction and the comparison metrics are described in more detail in the following section.

### In Vivo Experiments

The same acquired data were used retrospectively to produce a 2× undersampled non‐motion corrected (NMC) reconstruction (2× undersampling in radial direction, no undersampling in angular direction), a 2× undersampled 5 mm self‐gated reconstruction (2× undersampling in radial direction, no undersampling in angular direction), and a highly undersampled TV‐GMD reconstruction (2× undersampling in radial direction, 1.8× undersampling in angular direction, on average). This resulted in a total of 256, 414 ± 147 and 160 ± 37 acquired profiles for the NMC, gated and TV‐GMD reconstructions.

The following binning parameters were used: α_max_ = 13.75° (determined according to the previous section); *w_max_* = 5 mm (same value as the gating window); *GE_min_* = 80% (set to remove outliers in the respiratory cycle). Furthermore, to avoid remaining undersampling artifacts in the final motion corrected reconstruction a maximum undersampling factor (*R_max_*) of 4 was used. Therefore, the proposed method was set to reconstruct a minimum of 128 radial profiles, equivalent to 2× angular undersampling and 2× radial undersampling. This resulted in data being grouped into three to five bins (varying per volunteer), with bin undersampling factors ranging from 5.2× to 18.9× (average of 9.5×). The motion corrected datasets were undersampled from a minimum of 2.1× to a maximum of 4× (average of 3.6×). Table 1 presents values for undersampling factors, gating efficiency, acquisition time, number of acquired profiles, number of profiles per bin, number of bins, and bin window sizes. The respiratory bins were reconstructed with TV‐SENSE. The initial *l*_2_‐norm reconstruction ran with 10 iterations, with **λ_s_** = 0 and **λ_t_** = 0. This result was used as a starting estimate for the reconstruction with **λ_s_** = 0.2 and **λ_t_** = 0.1 (corresponding to 9.6 × 10^−6^ and 4.8 × 10^−6^, respectively, in terms of the average ǁ.ǁ**_∞_** norm of the images), three iterations. The reconstructed bins were registered to estimate the motion for the TV‐GMD reconstruction. The initial data consistency GMD reconstruction ran with five iterations, **λ_s_** = 0. The following TV‐GMD reconstruction used **λ_s_** = 0.0001 (corresponding to 4.8 × 10^−9^, in terms of the average ǁ.ǁ**_∞_** norm of the images) and five iterations. Additionally, data were reconstructed with GMD (4 iterations) for additional comparison with TV‐GMD. It took approximately 1.5 h to reconstruct bins (TV‐SENSE) and an additional 1.5 h for the motion compensated reconstruction (TV‐GMD) on a 12‐core implementation. The gated and nonmotion corrected images were reconstructed with iterative SENSE 23, both taking approximately 5 min to reconstruct. Coil sensitivities were estimated from a reference scan. All images were reconstructed using Matlab (The MathWorks, Natick, MA).

**Table 1:** Acquisition and reconstruction information for non‐motion corrected (NMC), gated, GMD and TV‐GMD reconstructions.

Acquisition and reconstruction	NMC	Gated	GMD	TV‐GMD
Number of bins	1	1	3‐5	3‐5
Gating/Bin windows (mm)	11.1 ± 3.5	5.00	2.76 ± 1.1	2.76 ± 1.1
Number of profiles per bin	256	256	49 ± 16	49 ± 16
Number of profiles acquired	256	414 ± 147	160 ± 37	160 ± 37
Number of profiles reconstructed	256	256	148 ± 37	147 ± 37
Acquisition time (s)	62.98	101.79 ± 36.21	39.36 ± 9.13	39.36 ± 9.13
Gating efficiency (%)	100	67 ± 15	93 ± 7	93 ± 7
Undersampling factor	2	2	3.6 ± 0.5	3.6 ± 0.5

To observe the effect of undersampling in the final motion corrected reconstruction, data from two representative volunteers were reconstructed with the proposed method with undersampling factors of 4×, 3×, 2×, and 1×, using the same estimated motion as described above.

The proposed TV‐GMD approach was compared with the gated reconstruction by means of liver sharpness, gradient entropy, visual image scoring and ranking 31. The liver sharpness and gradient entropy metrics were computed on a set of 10 coronal slices. To compute liver sharpness, twenty‐five 1D profiles were manually selected across the liver–lung interface. The sharpness measure for each profile was obtained by the maximum gradient normalized to the maximum intensity, similar to Botnar et al 32. Gradient entropy has previously been used in motion correction as an optimization cost function that favors distinct boundaries and reduced artifacts and was ranked 1 of 24 image quality metrics studied 33. Here we use local gradient entropy as an image quality metric, similar to Vuissoz et al 34. The total gradient entropy of the image is given by the mean of the local gradient entropies. All liver sharpness and gradient entropy values were normalized to the reference gated values. We present the gradient entropy results inverted to allow easier comparison with the other metrics (the higher the metric the better the image). Two experts (a radiologist with 14 years of experience in MRI and an image processing researcher with 18 years of experience in MRI) were asked to “score the sharpness of the main boundaries and features of the images” on a scale of 0 (extreme blurring) to 4 (no blurring) and “rank the overall image quality based on existing artifacts, ghosting and blurring” from 1 (worst) to 4 (best). Statistical significance of gradient entropy and liver sharpness was evaluated using a paired t‐test (*P*‐value of 1%); statistical significance of expert sharpness score and quality rank was evaluated using a Wilcoxon signed rank test (*P*‐value of 1%).

## RESULTS

### Simulations

**Figure 2:**
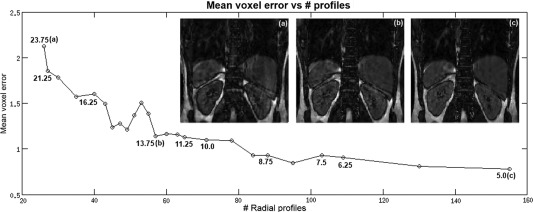
Plot of the mean motion field error as a function of the number of radial profiles used for bin reconstruction. Several α values are marked in the graph. Respiratory bin reconstruction for the volunteer studied for 3 different α values are shown: **(a)** α = 5°, **(b)** α = 13.75°, and **(c)** α = 16.25°. For α ≤ 13.75°, motion field errors are kept below 1.1 voxels.

Figure 2 shows the mean voxel error in the registration deformation field decreasing with the increasing number of radial profiles. Angular gaps values (α) resulting for different number of radial profiles are displayed throughout the graph. For values lower than α = 13.75° the mean voxel error in the motion fields remains below 1.1 voxels. For α values larger than 13.75° the error in the motion fields start increasing significantly. Based on this simulation, we chose to set α_max_ = 13.75°.

### Comparison with Image Based Motion Compensation

**Figure 3:**
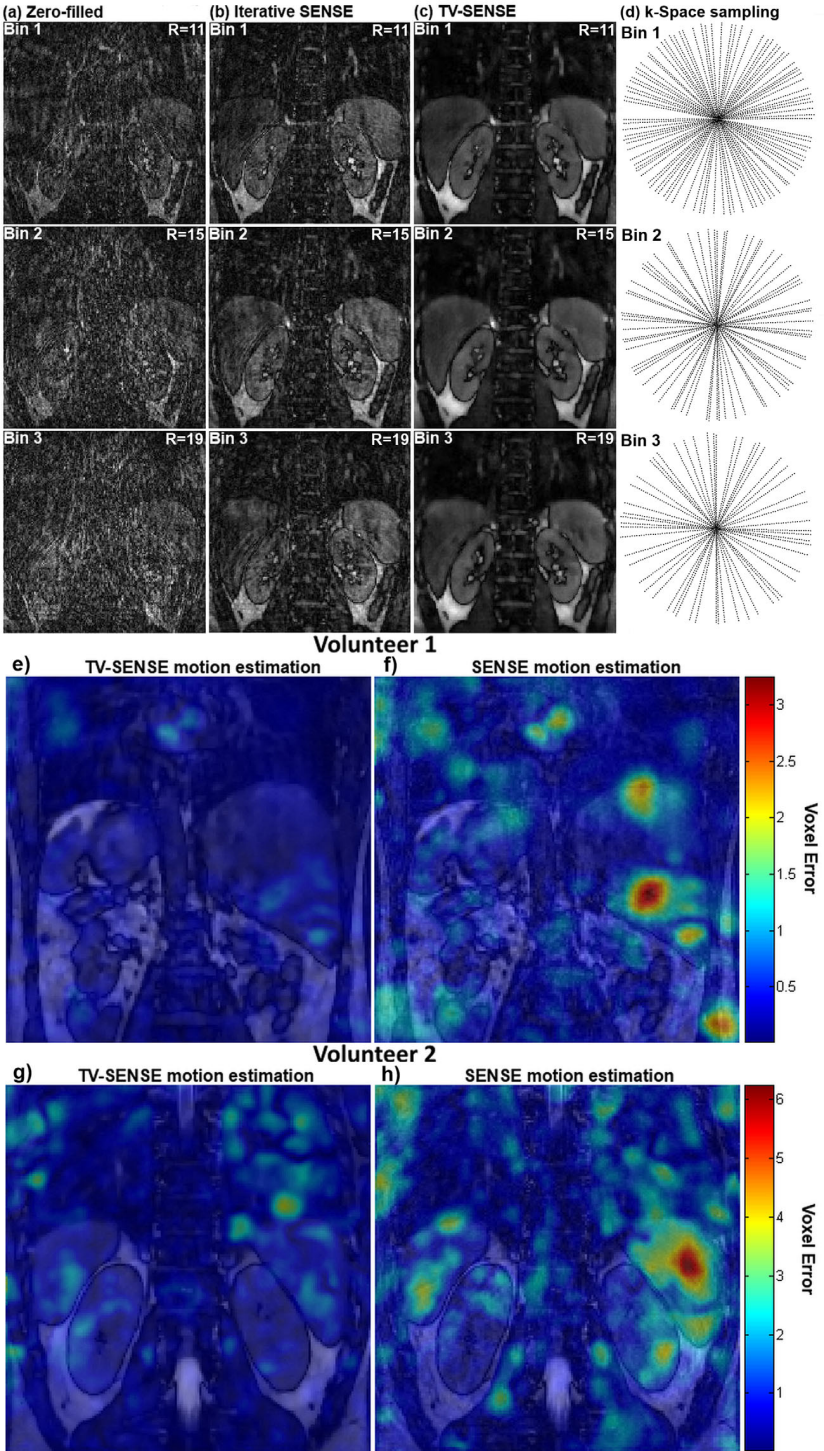
Reconstructions of bins 1, 2, and 3 with 46, 33 and 27 radial profiles (corresponding to undersampling factors R of 11, 15 and 19, respectively). **a**: Zero‐filled reconstructions. **b**: Iterative SENSE used in 16, significant aliasing remains. **c**: TV‐SENSE, most aliasing is eliminated. **d**: k‐Space sampling locations in the phase encoding plane for bins 1, 2, and 3. **e**–**h**: Motion estimation errors (in voxel units) between neighboring bins for TV‐SENSE and iterative SENSE bin reconstructions for two volunteers. Color coded motion estimation errors are overlaid on the anatomical reconstruction. Iterative SENSE motion estimation (f–h) shows increased local errors, whereas TV‐SENSE provides more reliable motion estimation (e–g).

Figures 3a–c show bin images of volunteer 2 for a zero‐filled reconstruction, iterative SENSE and TV‐SENSE for resulting undersampling factors (R) of ≈11×, ≈15×, and ≈19× for bins 1, 2, and 3, respectively. Iterative SENSE reconstruction, as used in Buerger et al 16, shows residual artifacts (Fig. 3b). These artifacts are significantly reduced with a TV‐SENSE reconstruction, while only introducing minor blurring (Fig. 3c).

The improvement in motion estimation accuracy with the proposed TV‐SENSE in comparison to previously shown iterative SENSE can be seen in Figures 3e–h, for two representative volunteers. Image registration of SENSE reconstructions can produce local errors (yellow‐red regions in Figures 3f,h) as the LREG algorithm attempts to register aliasing artifacts. TV‐SENSE removes most of these artifacts, enabling reliable motion estimation (corresponding blue‐green regions in Figures 3e,g).

**Figure 4:**
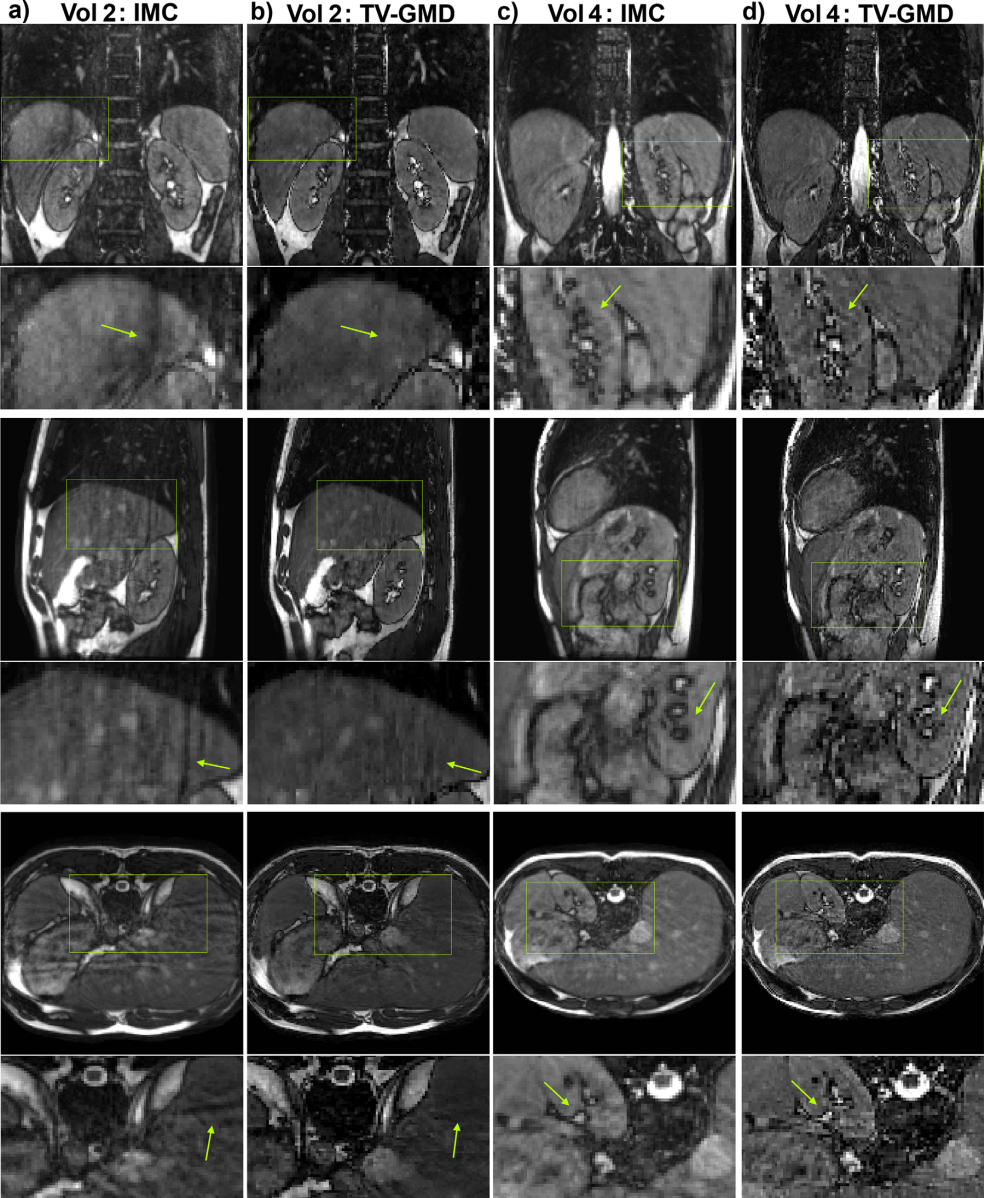
Coronal (top), sagittal (middle) and axial (bottom) slices for volunteers 2 and 4 (including zoom‐in images, arrows point out some main differences). **a**: Image based motion correction (IMC) for volunteer 2 with 4× undersampling at 100% gating efficiency. Residual aliasing may be seen in all slice orientations. Additionally, some blurring is introduced by IMC. **b**: Proposed TV‐GMD for volunteer 2 with 4× undersampling at 100% gating efficiency. Residual aliasing is reduced and image structures appear sharper when compared with IMC. **c**: IMC for volunteer 4 with 4× undersampling at 100% gating efficiency. Residual aliasing is not significant in this volunteer, but the IMC still introduces additional blurring to the image. **d**: TV‐GMD for volunteer 4 with 4× undersampling at 100% gating efficiency. A sharper reconstruction is obtained with the TV‐GMD.

Figure 4 shows multiple slice orientations of two volunteers for image‐based motion compensation (IMC) and TV‐GMD reconstructions. If undersampling artifacts from different motion states fail to cancel out, residual aliasing may arise in IMC as shown in Figure 4a for volunteer 2. This aliasing is significantly reduced with the TV‐GMD (Fig. 4b). Results for volunteer 4 using IMC (Fig. 4c) present less residual aliasing, but additional blurring is introduced (which can be seen in both volunteers). In contrast, the TV‐GMD corrects motion without introducing this additional blurring (Fig. 4d). TV‐GMD and IMC presented liver sharpness values of 1.18 and 0.98, respectively, and gradient entropy values of 1.00 and 0.98, both statistically different with a *P*‐value of 1%.

### In Vivo Experiments

**Figure 5:**
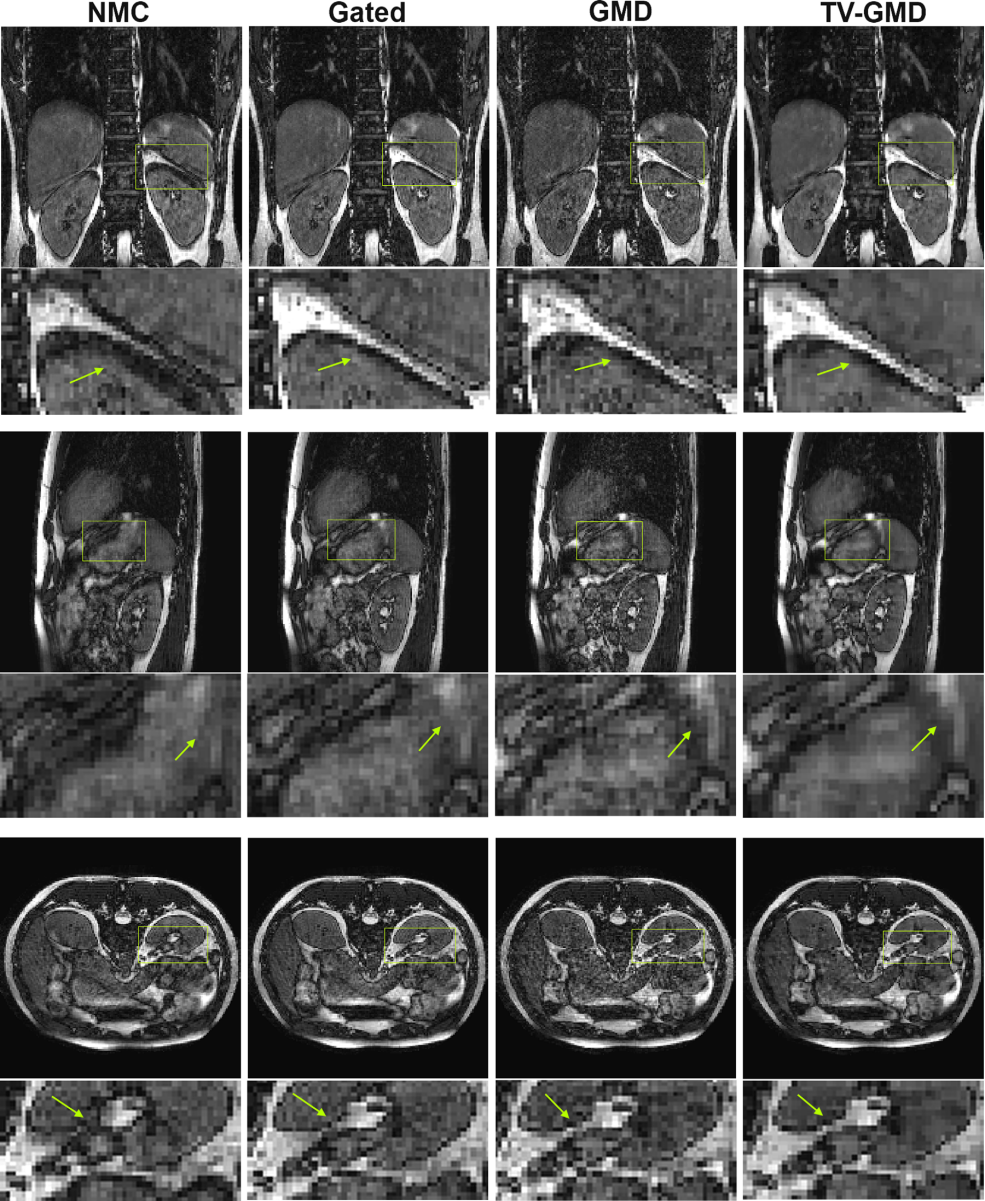
Coronal (top), sagittal (middle) and axial (bottom) slices for volunteer 1 with maximum respiratory amplitude of 14.8 mm (including zoom‐in images, arrows point out some main differences). **NMC (non‐motion corrected):** 2× undersampled at 100% gating efficiency. Several structures in the image are corrupted by motion. **Gated:** 2× undersampled at 60% gating efficiency. Most structures are sharper than the NMC. **GMD:** 3.5× undersampled at 80% gating efficiency. The GMD is sharper than the NMC, but presents remaining undersampling artifacts. **TV‐GMD:** 3.5× undersampled at 80% gating efficiency. The total variation regularization improves undersampled reconstruction at the expense of minor blurring.

**Figure 6:**
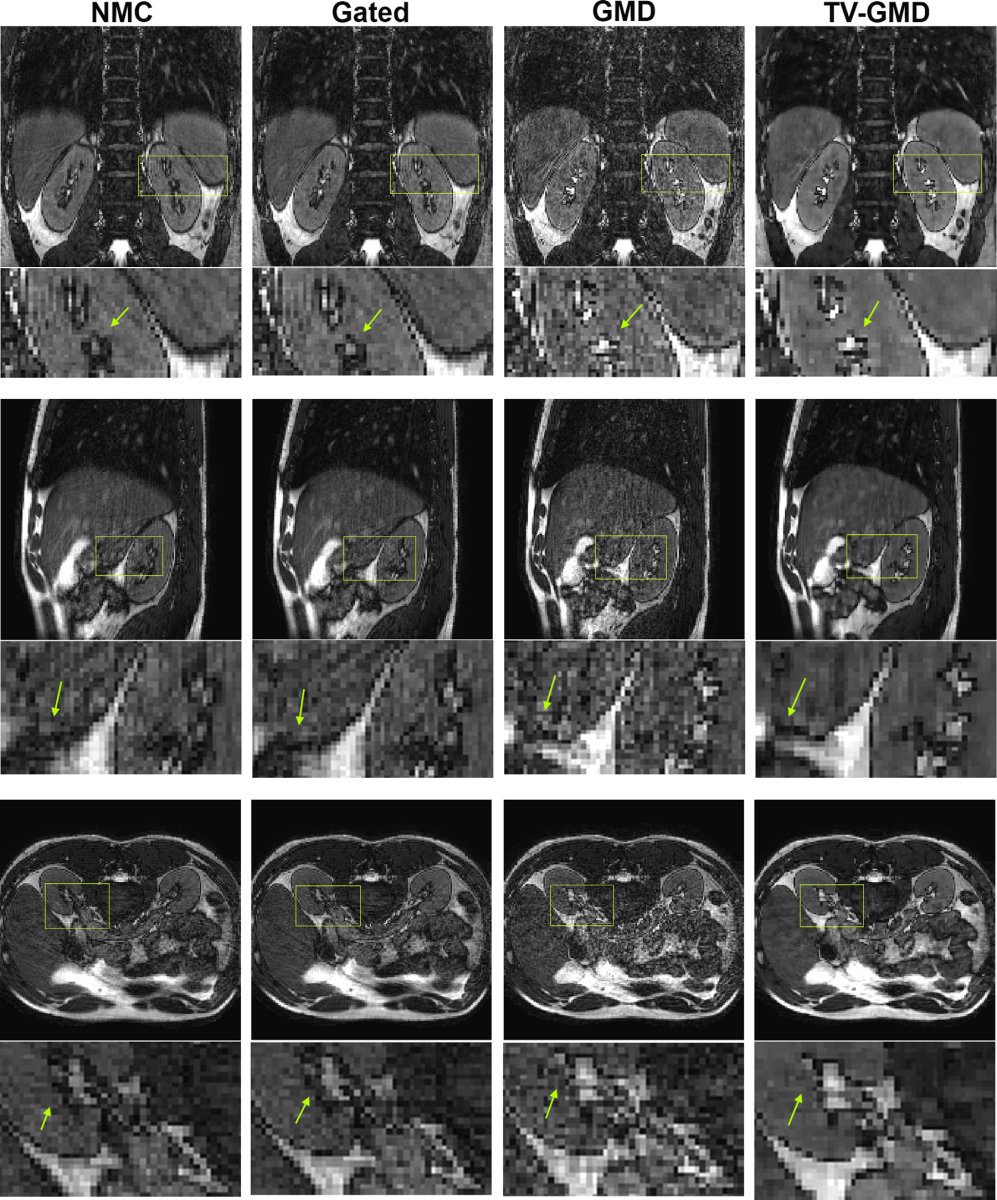
Coronal (top), sagittal (middle) and axial (bottom) slices for volunteer 2 with maximum respiratory amplitude of 8.2 mm (including zoom‐in images, arrows point out some main differences). **NMC (non‐motion corrected):** 2× undersampled at 100% gating efficiency. Some blurring is visible in image structures and the liver–lung border. **Gated:** 2× undersampled at 77% gating efficiency. There is no significant improvement, as the gated reconstruction uses a 5 mm window. **GMD:** 4× undersampled at 96% gating efficiency. The high undersampling creates a strong noise‐like aliasing. **TV‐GMD:** 4× undersampled at 96% gating efficiency. The total variation regularization improves GMD undersampled reconstruction at the expense of some minor blurring, despite using only 128 radial profiles.

Figure 5 shows multiple slice orientations for the nonmotion corrected, gated, GMD and TV‐GMD reconstructions for volunteer 1. In Figure 5, it can be seen that TV‐GMD and gated reconstructions yield images of similar quality, correcting most ghosting and blurring present in the non‐motion corrected (NMC). The gated and NMC reconstructions have an undersampling factor of 2×, while the GMD and TV‐GMD resulted in an undersampling of 3.5× for this volunteer. Remaining artifacts from undersampling and noise amplification can be observed in the GMD reconstruction (Fig. 5). This effect is consistent across all volunteers and increases for higher undersampling factors. Comparison between GMD and TV‐GMD highlights how TV regularization improves the conditioning of the reconstruction. Figure 6 shows multiple slice orientations for volunteer 2, where the GMD and TV‐GMD reconstructions resulted in an undersampling of 4×, compared with 2× for the gated and NMC. The TV‐GMD presents a sharper reconstruction than the gated (Fig. 6), due to the fact that the resulting binning windows were smaller (2.76 mm average) than the gating window (5 mm). Note that undersampling artifacts are stronger for this case, but still can be reduced by TV regularization.

**Figure 7:**
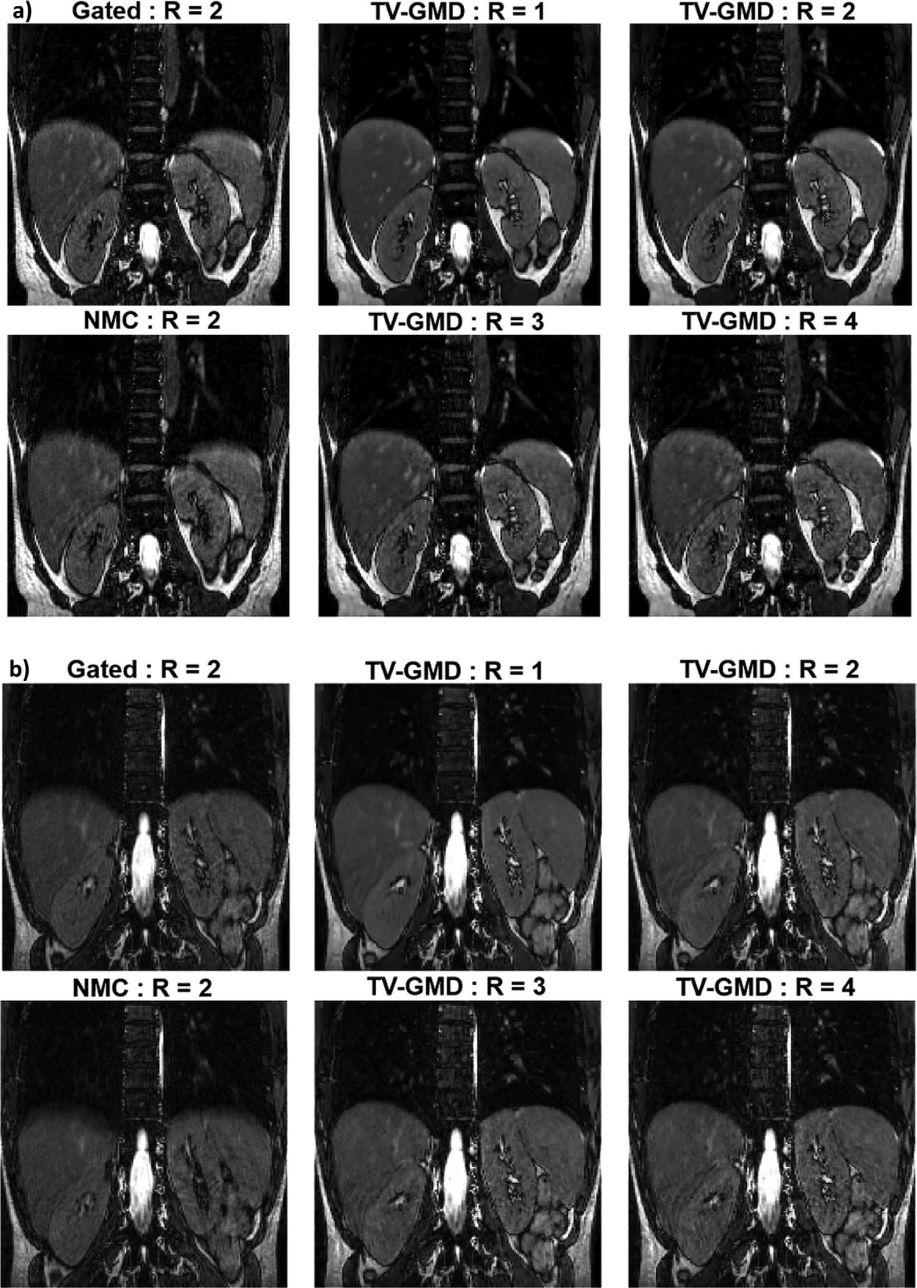
**a:** Coronal slices for volunteer 3 reconstructed with TV‐GMD at undersampling factors (R) of 4×, 3×, 2×, and 1×, corresponding to 128, 170, 256, and 512 radial profiles, respectively. The gated and nonmotion corrected (NMC) reconstructions have an undersampling factor of 2. A signal‐to‐noise improvement is visible as more data are used for the reconstruction. Vessel features benefit particularly from this additional data. **b**: Coronal slices for volunteer 4 reconstructed with TV‐GMD at undersampling factors (R) of 4×, 3×, 2×, and 1×, corresponding to 128, 170, 256, and 512 radial profiles, respectively. The gated and non‐motion corrected (NMC) reconstructions have an undersampling factor of 2. Small, low contrast features in the image become better defined with the TV‐GMD reconstruction and benefit from the lower undersampling factors.

Reconstructions for the TV‐GMD at various undersampling factors in two volunteers are shown in Figures 7a,b. As expected, undersampling artifacts decrease with increasing data used. The proposed method is flexible in the choice of the maximum undersampling factor of the reconstructed image which may be useful for clinical applications that require specific signal‐to‐noise ratios.

**Figure 8:**
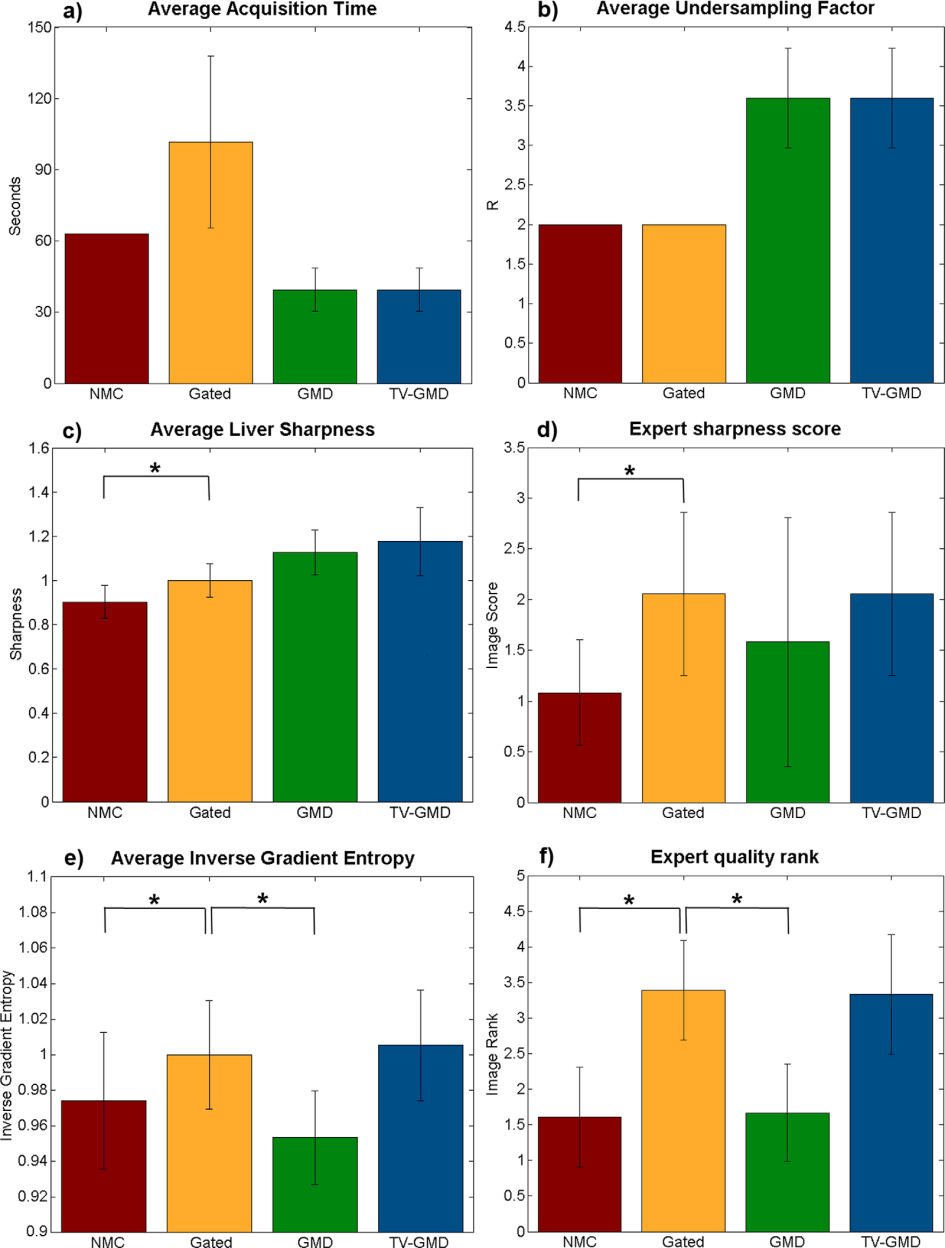
Bar plots comparing the performance of the non‐motion corrected (NMC), gated, GMD and proposed TV‐GMD reconstructions in terms of average acquisition time **(a)**, average undersampling factor **(b)**, average liver sharpness **(c)**, expert sharpness score (0: extreme blurring to 4: no blurring) **(d)**, average (inverse) gradient entropy **(e)**, and expert overall quality rank (1: worst to 4: best) **(f)**. Statistically different results with a *P*‐value of 1% are marked with (*).

Bar plots of the average acquisition times, undersampling factors, gradient entropies, liver sharpness, sharpness score, and image quality rank over all nine volunteers are shown in Figure 8. When comparing with the gated reconstruction, the TV‐GMD reconstruction reduces the average scan time from 101 to 34 s. The gradient entropy indicates the TV‐GMD and gated reconstructions have the best image quality (1.00 and 1.00, respectively), significantly better than NMC (0.97) and GMD (0.95). Image rank from visual assessment agreed with gradient entropy, marking TV‐GMD (3.33) just below the gated (3.39), significantly better than NMC (1.61) and GMD (1.67). Image sharpness score from visual assessment yielded similar values between the TV‐GMD (2.22) and gated (2.44), differentiating them from the NMC (1.17). Liver sharpness measures were not as sensitive as expert sharpness evaluation, but presented a similar behavior, marking TV‐GMD as the best (1.18), followed by GMD (1.12) and gated (1.00), significantly better than NMC (0.90).

## DISCUSSION

The proposed approach accelerates the acquisition by means of undersampling and 3D nonrigid motion correction, reducing the average scan time by 2.6× when compared with a gated acquisition. Here, motion is estimated (TV‐SENSE) from highly undersampled respiratory resolved images and corrected in the reconstruction (TV‐GMD) of the undersampled final data set. Gradient entropy and image quality ranking present similar values for the TV‐GMD and gated reconstructions, distinguishing them as superior to the NMC and GMD. The noise‐like aliasing present in the GMD reconstruction is responsible for the worse values in gradient entropy. The liver sharpness and expert sharpness score show the TV‐GMD removes motion‐induced blurring (visible in the NMC) to a similar level as the gated reconstruction. In cases of uniform breathing the TV‐GMD achieves sharper reconstructions than the gated as it uses binning windows (2.76 mm average) smaller than the gated window (5 mm).

This experiment was carried out retrospectively to compare different approaches, but the decision to stop a running acquisition once enough data have been acquired could be performed prospectively. This process requires a 1D FFT and a cross‐correlation (to obtain the self‐navigation signal), followed by the adaptive binning algorithm, taking less than 3 ms to compute. One limitation of the proposed method is the 3 hour reconstruction time. Reconstruction times increase with both the amount of data used and the number of bins. The bottleneck in these reconstructions is the nonuniform Fourier transform, which may be accelerated by GPU implementations 35. Another solution to this problem would be to use Cartesian trajectories with similar properties to the G‐RPE, such as G‐CASPR 12, 36 or VDRad 37.

Note that the proposed approach requires a superior–inferior readout for self‐navigation and alternative navigation strategies will be needed for trajectories with readouts in other directions. Another limitation is error propagation from motion estimation. If the estimated motion is not accurate, this can lead to artifacts in the TV‐GMD reconstruction (results not shown here). Here we force every bin to respect α_max_ to guarantee accurate and reliable motion estimation. The proposed method is limited to inter‐bin motion correction, thus a significant fraction of k‐space remains uncorrected in the form of intra‐bin motion. In this work, we perform motion compensated reconstructions with undersampling factors up to 4×. At high undersampling factors, over‐regularization with total variation may create piecewise smoothing artifacts therefore an appropriate selection of a regularization parameter is needed.

It has been shown that GMD is capable of nearly perfect motion correction when the motion fields are known exactly 13. Thus, future improvements should target the motion estimation. First, self‐navigation may be improved by using multiple coil information 38, as opposed to the single coil approach used here. Second, the number of bins may be increased by lowering the bin window size, *w_max_*. This approach requires longer acquisitions and additional reconstruction time. However intra‐bin motion and total variation artifacts of the motion corrected images should be reduced. Third, bin quality may be improved by introducing pseudo‐random sampling in the radial direction, which may improve both motion estimation and motion compensated reconstruction. The value α_max_ was determined from a single volunteer study and although it provided successful reconstructions for all nine volunteers, further investigation may provide a more optimal setting.

**Figure 9:**
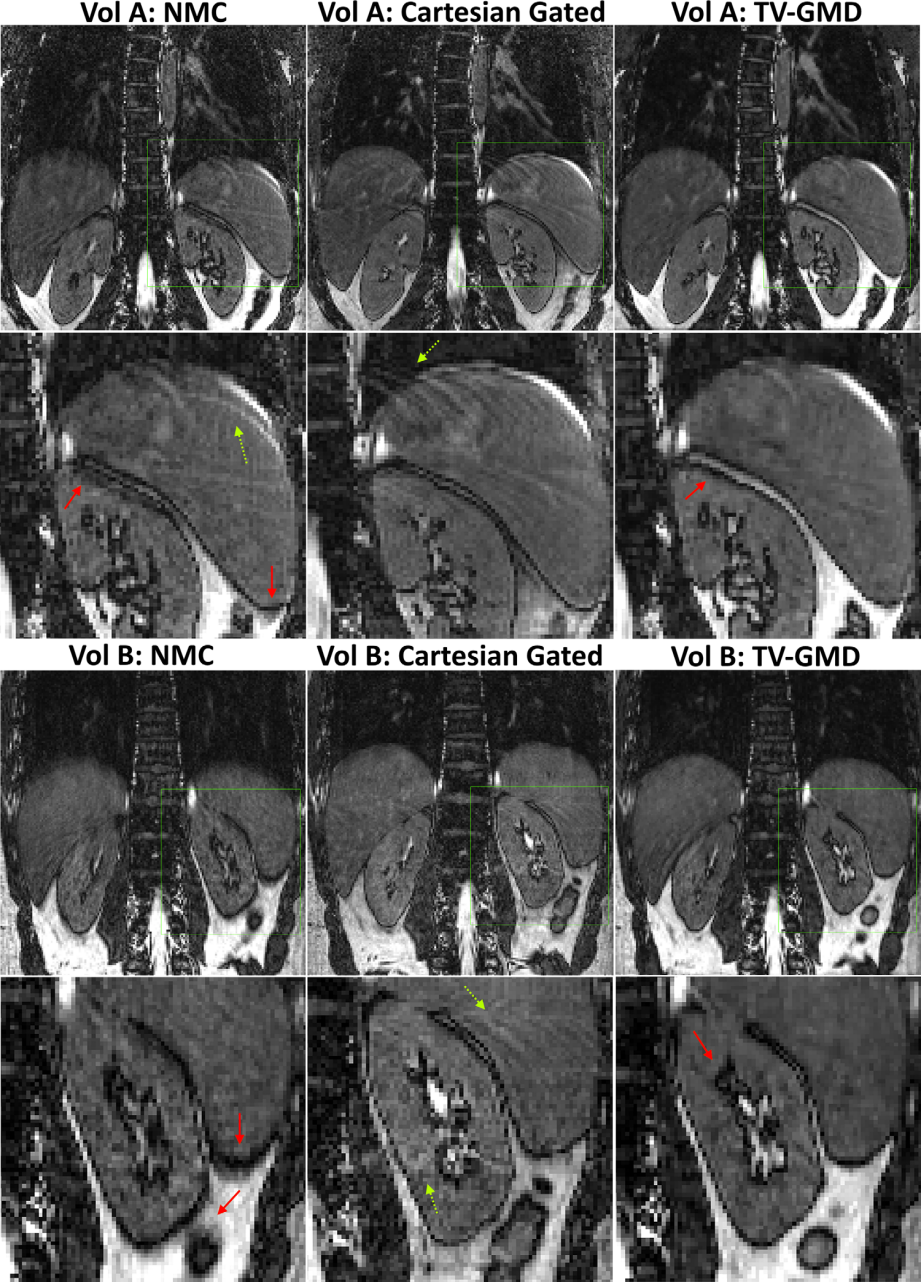
Coronal slices for a nonmotion corrected (NMC) G‐RPE 2× radially undersampled, Cartesian navigator gated (5 mm) 2× undersampled and TV‐GMD G‐RPE 2× radially undersampled for two volunteers. The Cartesian gated and TV‐GMD approaches compensate motion to a comparable degree. Residual motion is still present in the Cartesian gated (ghosting, yellow dotted arrows) and TV‐GMD (minor blurring, red full arrows).

A preliminary study was performed to compare the proposed approach with a Cartesian gated acquisition. 2× undersampled Cartesian navigator gated (5 mm) and G‐RPE datasets were acquired on two healthy volunteers using the same parameters as before. Figure 9 shows coronal slices for a non‐motion compensated (NMC) G‐RPE 2× radially undersampled, Cartesian gated 2× undersampled (AP direction) and TV‐GMD G‐RPE 2× radially undersampled for two volunteers. These initial results show that Cartesian gated and TV‐GMD remove most motion artifacts present in the NMC images. However, residual motion in the Cartesian gated creates coherent ghosting, whereas in TV‐GMD it creates minor blurring. Further studies in patients will be needed for clinical validation.

## CONCLUSIONS

A motion compensated reconstruction framework for accelerated 3D abdominal imaging has been presented. The proposed approach does not require additional training data or external sensors. Motion is estimated from highly undersampled data and incorporated into the reconstruction. In vivo results demonstrate the potential of the proposed method to provide similar image quality as a gated acquisition while reducing scan times by a factor of 2.6.
